# Association Between Glycemic Variability and Bone Turnover and the Sema3A Signaling Pathway in Postmenopausal Women with Type 2 Diabetes Mellitus

**DOI:** 10.3390/metabo16100723

**Published:** 2026-09-28

**Authors:** Burcu Cosgun Bora, Sertas Erarslan

**Affiliations:** Department of Internal Medicine, Faculty of Medicine, Kütahya Health Sciences University, Kutahya 43500, Türkiye; cosgun1075@gmail.com

**Keywords:** glycemic variability, bone turnover, semaphorin 3A, type 2 diabetes mellitus, postmenopause

## Abstract

**Highlights:**

**What are the main findings?**

- Short-term glycemic variability was independently associated with bone turnover and circulating Sema3A-related biomarkers in postmenopausal women with T2DM, regardless of mean glycemia: the coefficient of variation (CV) with the bone formation/resorption balance (P1NP/CTX-1), and the daily peak-to-trough amplitude (Δ glucose) with NRP1 and the NRP1/PLXNA1 and SEMA3A/NRP1 ratios.
- HbA1c-based glycemic control groups did not differ in bone turnover markers, Sema3A pathway components, or bone mineral density, whereas glycemic variability indices (SD, CV, Δ glucose) were consistently associated with these measures.

**What are the implications of the main findings?**

- Assessing bone health in postmenopausal women with type 2 diabetes may require attention to short-term glycemic variability, not just average glycemic control, even when bone mineral density appears normal.
- The NRP1/PLXNA1 and SEMA3A/NRP1 ratios are hypothesis-generating candidate biomarkers that warrant validation in longitudinal, multicenter studies before clinical application.

**Abstract:**

**Background:** This study evaluated relationships between glycemic variability, bone turnover markers, Sema3A pathway components, and bone mineral density in postmenopausal women with type 2 diabetes mellitus (T2DM). **Methods:** In this single-center cross-sectional study with prospective data collection, 126 postmenopausal women with T2DM performed capillary glucose measurements eight times daily for five days. Mean glucose, SD, CV, Δ glucose, and postprandial increment were calculated. Serum P1NP, CTX-1, SEMA3A, NRP1, and PLXNA1 were measured by ELISA, and P1NP/CTX-1, NRP1/PLXNA1, and SEMA3A/NRP1 ratios were derived. Bone mineral density was assessed by DXA. Associations were examined using Spearman correlation and multivariate regression adjusted for age, BMI, diabetes duration, vitamin D, PTH, insulin use, mean glucose, CV, and Δ glucose. **Results:** Bone turnover markers, Sema3A components, ratios, and T-scores did not differ across HbA1c-based groups (all *p* > 0.05). SD, CV, and Δ glucose correlated with several markers and ratios. CV was independently associated with P1NP/CTX-1 (B = −0.00174, *p* = 0.017); Δ glucose with NRP1 (B = −0.205, *p* = 0.013) and NRP1/PLXNA1 (B = −0.00023, *p* = 0.005); and both Δ glucose (B = 0.00035, *p* < 0.001) and mean glucose (B = −0.00022, *p* = 0.021) with SEMA3A/NRP1. **Conclusions:** Short-term glycemic variability, especially Δ glucose, was associated with bone turnover measures and exploratory Sema3A-related biomarkers independently of average glycemia; these associations may warrant consideration in future longitudinal studies of bone health in postmenopausal women with T2DM.

## 1. Introduction

Diabetes mellitus (DM) is a multisystemic metabolic disorder characterized by chronic hyperglycemia that affects carbohydrate, lipid, and protein metabolism and can lead to microvascular and macrovascular complications over time [1]. According to the 11th edition of the International Diabetes Federation (IDF) Diabetes Atlas, approximately 589 million adults aged 20–79 years were living with diabetes worldwide as of 2024, and this number is projected to reach 853 million by 2050 [2].

Type 2 diabetes mellitus (T2DM) accounts for approximately 90–95% of all diabetes cases and develops primarily against a background of insulin resistance and progressive beta-cell dysfunction [3]. Epidemiological studies have shown that although bone mineral density (BMD) is generally preserved or even increased in individuals with T2DM, fracture risk particularly for hip and vertebral fractures is increased [4]. This discrepancy between BMD and fracture risk is a key feature of the condition termed “diabetic osteopathy,” which is characterized more by impaired bone quality than by reduced bone mass [5]. Consistent with this, reduced levels of bone formation markers [procollagen type 1 N-terminal propeptide (P1NP) and osteocalcin] and the bone resorption marker [C-terminal telopeptide of type 1 collagen (CTX-1)] have been reported in patients with T2DM, a pattern suggested to reflect low-turnover diabetic bone disease [6]. In addition, some antidiabetic treatments particularly thiazolidinediones have shown adverse effects on bone health, whereas a fracture-risk signal associated with canagliflozin has not been consistently confirmed across studies [7,8,9].

HbA1c, one of the principal indicators used to assess glycemic control, reflects long-term average glycemia but does not adequately capture within-day and day-to-day glucose variability. Recurrent glucose fluctuations have been shown in experimental studies to increase oxidative stress and endothelial dysfunction more markedly than sustained hyperglycemia [10,11]. Glycemic variability (GV) has also been shown to be associated with diabetic bone disease independently of HbA1c [12]. However, studies directly evaluating the relationship between GV and bone turnover markers and mineralization parameters are limited, and available data are predominantly derived from cross-sectional studies.

One of the pathways that has recently emerged as important in the molecular regulation of bone turnover is the signaling axis formed by semaphorin 3A (Sema3A)—initially described as an axonal guidance protein—together with its receptor components neuropilin-1 (NRP1) and plexin-A1 (PLXNA1). Sema3A, secreted by osteoblasts, has been shown to support osteoblast differentiation and suppress osteoclast differentiation via the NRP1–PLXNA1 receptor complex, thereby contributing to the preservation of bone mass [13]. In experimental diabetes models, changes in the Sema3A pathway under high-glucose conditions have been reported to be accompanied by reduced osteogenic differentiation and impaired bone microarchitecture [14]. However, studies evaluating this pathway in humans—particularly in the context of glycemic variability—remain extremely limited.

Accelerated bone loss due to estrogen deficiency in the postmenopausal period renders women more susceptible to fracture [15]. The addition of T2DM-specific low bone turnover and impaired bone quality to this process makes postmenopausal women with T2DM a clinically important population for investigating diabetic bone fragility.

In this context, the study’s central hypothesis was that increased glycemic variability in postmenopausal women with T2DM is associated with changes in P1NP and CTX-1 levels and with differences in circulating components of the Sema3A pathway, and that these biochemical differences may be observable even in the absence of marked changes in BMD as measured by DXA.

Accordingly, the present study aimed to evaluate the relationships between glycemic variability indices—derived from frequent home-based capillary glucose measurements in postmenopausal women with T2DM—and bone turnover markers, circulating Sema3A pathway components, and BMD as measured by DXA. In doing so, the study sought to provide clinical data on the possible biochemical links between glycemic variability and diabetic bone metabolism.

## 2. Materials and Methods

### 2.1. Study Design and Ethical Approval

This single-center, cross-sectional, observational study with prospective data collection was conducted among postmenopausal women with type 2 diabetes mellitus (T2DM) who presented to the Internal Medicine Outpatient Clinic of Kütahya City Hospital from 1 September 2025, to 28 February 2026.

The study protocol was approved by the Non-Interventional Clinical Research Ethics Committee of Kütahya Health Sciences University Faculty of Medicine (Decision No: 2025/09-45; date: 14 July 2025).

The study was conducted in accordance with the principles of the Declaration of Helsinki, and written informed consent was obtained from all participants before enrollment.

### 2.2. Sample Size

Sample size was calculated a priori using G*Power 3.1.9.7, based on an independent-samples *t*-test comparing the primary bone turnover index (P1NP/CTX-1 ratio) between glycemic-variability subgroups. Because no prior pilot data were available for this specific biomarker panel, a conservative medium-to-large effect size (Cohen’s d = 0.63) was assumed; with a two-sided α of 0.05 and 80% power, at least 109 participants were required. This subgroup-based calculation served as an approximate a priori planning exercise to guide recruitment and was not intended to formally power the continuous correlation and multivariable regression analyses that constitute the primary analytic approach for this study (Section 2.5 and Section 2.10); the assumed effect size was a conservative estimate chosen in the absence of pilot data for this specific biomarker panel. Accounting for potential participant dropout and missing measurements, enrollment of 130–140 patients was planned. Participant flow, including reasons for exclusion, is summarized in Figure 1; of the 139 women initially followed, 13 were excluded because of missing data in at least one required domain (12 of these 13 in more than one domain), leaving 126 women in the final complete-case analysis. No imputation was performed. Baseline demographic and metabolic characteristics did not differ significantly between included and excluded patients (age, T2DM duration, BMI, fasting glucose, lipid profile, insulin use; all *p* > 0.05), except that excluded patients had a lower prevalence of hypertension (30.8% vs. 74.6%, *p* = 0.002) and a higher prevalence of coronary artery disease (46.2% vs. 17.5%, *p* = 0.024) (Supplementary Table S7).

### 2.3. Participants

Women aged 50–70 years who had been followed with a diagnosis of T2DM for at least five years and who had been postmenopausal for at least five years were included. Postmenopause was defined as at least 12 months of amenorrhea in the absence of any other physiological or pathological cause. Additional inclusion criteria were a body mass index (BMI) of 20–30 kg/m^2^ and having undergone dual-energy X-ray absorptiometry (DXA) for BMD assessment within the one month preceding glycemic evaluation.

Patients with a BMI < 20 or >30 kg/m^2^ were excluded to minimize the potential confounding effect of body weight on bone metabolism. Patients receiving bisphosphonates, denosumab, teriparatide, or hormone replacement therapy; those with chronic kidney disease (GFR < 60 mL/min), primary or secondary hyperparathyroidism, active malignancy, or another rheumatologic or systemic disease that could affect bone metabolism; and those using systemic corticosteroids, antiepileptic drugs, or other medications with a significant effect on bone metabolism, as well as patients with anemia or polycythemia, were also excluded.

### 2.4. Collection of Clinical and Laboratory Data

Participants’ age, duration of T2DM, comorbidities, and current medications were recorded through face-to-face interviews and medical records. Antidiabetic treatments were further classified by drug class. Height and body weight were measured using standard methods. BMI was calculated as body weight in kilograms divided by height in meters squared.

Fasting plasma glucose (FPG), hemoglobin A1c (HbA1c), 25-hydroxyvitamin D, parathyroid hormone (PTH), total cholesterol, LDL cholesterol, HDL cholesterol, triglycerides, urea, creatinine, aspartate aminotransferase (AST), alanine aminotransferase (ALT), alkaline phosphatase (ALP), uric acid, thyroid-stimulating hormone (TSH), calcium, phosphorus, and complete blood count results obtained at presentation were retrieved from the hospital information management system. All routine laboratory analyses were performed at the hospital’s central laboratory using standard methods.

### 2.5. Home Capillary Glucose Monitoring and Assessment of Glycemic Variability

All participants were provided with the same model of capillary glucose meter (Accu-Chek Performa; Roche Diagnostics, Mannheim, Germany) and compatible test strips, which meet the analytical performance requirements of ISO 15197:2013 [16]. Participants received hands-on training on hand hygiene before testing, device operation, storage of test strips, and recording of results.

Participants were asked to perform capillary fingerstick glucose measurements eight times daily—at 07:00, 09:00, 12:00, 14:00, 18:00, 20:00, 23:00, and 03:00—for five consecutive days. Measurements at 07:00, 12:00, and 18:00 were taken before breakfast, lunch, and dinner, respectively, while those at 09:00, 14:00, and 20:00 were taken two hours after the start of the corresponding meal. The 23:00 measurement assessed glucose levels before sleep, and the 03:00 measurement was used to evaluate the nocturnal glucose profile. No changes were made to participants’ antidiabetic treatment during the five-day monitoring period.

A total of 40 capillary glucose measurements were targeted for each participant over the five-day monitoring period. The postprandial increment (Equation 3) was calculated separately for each of the three main meals (breakfast, lunch, dinner) on each of the five monitoring days, yielding up to 15 values per participant; the mean of these values was used as the mean postprandial glucose increment. From these measurements, mean glucose, standard deviation (SD), coefficient of variation (CV), Δ glucose, and mean postprandial glucose increment were calculated.

CV (%) = SD/mean glucose × 100(1)

Δ glucose = Σ (daily maximum glucose − daily minimum glucose)/number of monitoring days(2)

Postprandial increment = 2 h postprandial glucose − pre-meal glucose(3)

Mean glucose, SD, CV, Δ glucose, and mean postprandial increment were treated as continuous variables in the primary analyses. Δ glucose (Equation 2) reflects the observed daily peak-to-trough amplitude across the eight scheduled measurements per day; because sampling was limited to these fixed time points, it may not capture the true 24 h glucose maximum or minimum, and this term is used throughout the manuscript with that meaning. Only participants with a fully complete set of 40 scheduled measurements were included in the primary analysis (*n* = 126 of 139); eight participants were excluded specifically because of incomplete home-monitoring records (Section 2.2), and the number and distribution of missing or invalid individual readings before this threshold was applied were not separately logged in the source dataset and therefore cannot be reported. Meal timing was standardized by protocol, with measurements scheduled at fixed clock times before and after each main meal (as above); daily physical activity was not standardized or restricted during the monitoring period, and its potential influence on glucose readings cannot be excluded. Device or transcription errors were minimized through structured training (above) and manual review of diary sheets by study staff, but a formal, pre-specified error-adjudication protocol was not implemented; this should be considered a limitation of the home-monitoring approach.

### 2.6. Assessment of Bone Mineral Density

Bone mineral density was measured at the lumbar spine (L1–L4), femoral neck, and total femur using an FDX Visionary-DR DXA device (Fujifilm/DMS Imaging, Gallardon, France) within the one month preceding glycemic assessment. BMD values were recorded in g/cm^2^, and standardized results were expressed as T-scores.

Postmenopausal women were classified according to World Health Organization T-score criteria: a T-score ≥ −1.0 was considered normal BMD, a T-score between −2.5 and −1.0 was considered osteopenia, and a T-score ≤ −2.5 was considered osteoporosis [17]. Lumbar spine, femoral neck, and total femur T-scores were also evaluated separately as continuous variables in statistical analyses.

### 2.7. Measurement of Bone Turnover and Sema3A-Related Biomarkers

Venous blood samples were collected in the morning (between 08:30 and 09:30) after at least eight hours of overnight fasting. Samples were centrifuged under appropriate conditions to separate serum, which was stored at −80 °C until analysis. Repeated freeze–thaw cycles were avoided. Venous blood sampling occurred at the initial study visit; at this same visit, participants received the capillary glucose meter and training (Section 2.5) and began the five-day home glucose monitoring period immediately afterward. This sequence—baseline blood draw followed by five days of home glucose monitoring—was identical for all participants.

Levels of the bone formation marker procollagen type 1 N-terminal propeptide (P1NP), the bone resorption marker C-terminal telopeptide of type 1 collagen (CTX-1), and the circulating Sema3A pathway components SEMA3A, NRP1, and PLXNA1 were measured using commercial kits based on the sandwich enzyme-linked immunosorbent assay method (BT Lab, Bioassay Technology Laboratory, Jiaxing, Zhejiang, China); catalog numbers, sensitivity, standard curve ranges, and intra-/inter-assay coefficients of variation are reported in Supplementary Table S5. These kits quantify soluble, circulating forms of SEMA3A, NRP1, and PLXNA1 present in serum; they do not distinguish membrane-bound receptor complexes and do not reflect local receptor density or intracellular signaling activity on osteoblasts or osteoclasts. Independent validation of these kits against international reference methods is limited (Supplementary Table S5), so throughout this manuscript these measures are referred to as circulating Sema3A-related biomarkers rather than as direct indices of Sema3A pathway signaling. All samples were run in the same assay batch and in duplicate; the mean of the two measurements was used in statistical analyses.

### 2.8. Exploratory Ratios Calculated for the Study

The P1NP/CTX-1 ratio was calculated to summarize the relative balance between bone formation and resorption in a single numerical index. Higher values indicate relatively higher P1NP relative to CTX-1, whereas lower values indicate the opposite.

Given that Sema3A exerts its effects through the binding co-receptor NRP1 and the signal-transducing receptor PLXNA1, the NRP1/PLXNA1 ratio was calculated to assess the relative balance between these receptor components. In addition, the SEMA3A/NRP1 ratio was included in the analyses to assess the relative balance between the ligand and its binding co-receptor.

These ratios were exploratory indices defined a priori in accordance with the study hypothesis and were not treated as validated measures of cellular signaling activity or established clinical indices. Ratio values may be disproportionately influenced by variation in either the numerator or denominator; this possibility is examined further in Section 4.3. Measurement units of the relevant biomarkers were harmonized before the ratios were calculated.

### 2.9. HbA1c Groups and Glycemic Variability Quartiles

Participants were divided into three glycemic control groups based on HbA1c level: Group 1, 5.0% ≤ HbA1c < 7.0% (31 ≤ HbA1c < 53 mmol/mol); Group 2, 7.0% ≤ HbA1c < 9.0% (53 ≤ HbA1c < 75 mmol/mol); and Group 3, HbA1c ≥ 9.0% (≥75 mmol/mol). Demographic and clinical characteristics, routine laboratory results, bone turnover markers, Sema3A pathway components, the exploratory ratios defined for this study, and regional T-scores were compared across these groups.

Glycemic variability indices were treated as continuous variables in the primary analyses. To examine possible graded relationships, SD, CV, and Δ glucose values were also divided into quartiles based on the sample distribution. These analyses were considered sample-based and exploratory.

### 2.10. Statistical Analysis

Statistical analyses were performed using IBM SPSS Statistics version 25.0 (IBM Corp., Armonk, NY, USA). The normality of continuous variables was assessed using the Shapiro–Wilk test and Q-Q plots; data were presented as mean ± SD or median (interquartile range) depending on distribution, and categorical variables as number (%). The three HbA1c-based groups were compared using one-way ANOVA for normally distributed variables and the Kruskal–Wallis test for non-normally distributed variables; Bonferroni-corrected pairwise comparisons were performed when overall tests were significant. Categorical variables were assessed using the chi-square test or Fisher’s exact test. Relationships between glycemic variability parameters (mean glucose, SD, CV, Δ glucose, postprandial increment) and bone turnover/Sema3A pathway markers and regional T-scores were examined using Spearman correlation (exploratory). Because this involved 55 correlations, raw *p*-values are reported alongside Benjamini–Hochberg false discovery rate (FDR)-adjusted q-values (Supplementary Table S4); this analysis was exploratory and hypothesis-generating rather than confirmatory of pre-specified primary or secondary endpoints.

To identify factors independently associated with each outcome, separate multivariate linear regression models were constructed for P1NP/CTX-1, P1NP, CTX-1, NRP1/PLXNA1, NRP1, PLXNA1, and SEMA3A/NRP1. Age, BMI, diabetes duration, 25(OH)D, PTH, insulin use, mean glucose, CV, and Δ glucose were entered simultaneously as covariates. Insulin use was selected as the primary treatment-related covariate because it is a recognized potential confounder of bone turnover with a plausible osteoanabolic effect [18], and because the ratio of participants to covariates limited how many medication variables could be added simultaneously to each of the seven regression models. To address residual confounding from other antidiabetic agents with reported skeletal effects, sensitivity analyses excluding pioglitazone users and adjusting for SGLT2 inhibitor use were performed separately and are reported in Supplementary Table S6d; other medication classes used in this cohort (e.g., metformin, sulfonylureas, DPP-4 inhibitors) are not consistently associated with altered bone turnover in the literature and were not modeled individually given sample-size constraints. SD was not included because it largely reflects the same dimension as CV, and postprandial increment was excluded because it represents overall postprandial exposure. Model assumptions (linearity, normality of residuals, homoscedasticity, Cook’s distance) and multicollinearity (VIF < 5, tolerance > 0.20) were assessed, with exact variance inflation factor and tolerance values reported in Supplementary Table S6; results are presented as B coefficients, 95% CI, and *p*-values, with model fit reported as R^2^/adjusted R^2^. Because the seven regression models were not corrected for multiplicity across models, these results should be interpreted as exploratory and hypothesis-generating, consistent with the framing adopted for the correlation and quartile analyses. To evaluate whether the reported glycemic-variability associations were independent of longer-term average glycemic exposure, a sensitivity analysis was additionally performed adding HbA1c (%) as a continuous covariate to each of the seven models (Supplementary Table S6f).

To examine graded, quartile-based patterns, SD, CV, and Δ glucose were divided into quartiles and compared using the Kruskal–Wallis test (with Bonferroni-corrected pairwise comparisons when significant). All tests were two-tailed; *p* < 0.05 was considered statistically significant.

### 2.11. Artificial Intelligence Use Statement

During the preparation of this manuscript, large language model-based artificial intelligence tools (Claude, Anthropic) were used solely for language editing and text revision. Study design, data collection, statistical analysis, and scientific interpretation were performed entirely by the authors. All content generated or revised with the assistance of artificial intelligence was reviewed and approved by the authors.

## 3. Results

The study included 126 postmenopausal women with T2DM. Patients were divided into three groups according to glycemic control level: Group 1 (HbA1c 5–7% [31–53 mmol/mol], *n* = 33), Group 2 (HbA1c 7–9% [53–75 mmol/mol], *n* = 47), and Group 3 (HbA1c ≥ 9.0% [≥75 mmol/mol], *n* = 46).

Table 1 presents the demographic and clinical characteristics of the patients. Age was similar across groups (*p* = 0.661). Diabetes duration was significantly longer in Group 3 than in Group 1 (*p* = 0.011). Body mass index did not differ across groups (*p* = 0.240). Fasting plasma glucose (*p* < 0.001) and HbA1c (*p* < 0.001) differed significantly across groups, as expected. No significant differences were found in lipid profile parameters (total cholesterol, LDL, HDL, triglycerides) across groups (all *p* > 0.05). Other laboratory values are presented in Supplementary Table S1, and the medication use profile in Supplementary Table S2.

Table 1 compares bone turnover markers, indices, and bone mineral density across glycemic control groups. No significant differences were found across groups in P1NP (*p* = 0.339), CTX-1 (*p* = 0.555), NRP1 (*p* = 0.167), PLXNA1 (*p* = 0.254), or SEMA3A (*p* = 0.518) levels. No significant differences were found in the P1NP/CTX-1 (*p* = 0.571), NRP1/PLXNA1 (*p* = 0.144), or SEMA3A/NRP1 (*p* = 0.928) ratios. L1–L4 (*p* = 0.455), femoral neck (*p* = 0.988), and total femur (*p* = 0.799) T-scores were similar across groups.

Table 2 presents the correlations between glycemic variability parameters and bone turnover markers, indices, and bone mineral density. Δ glucose was significantly correlated with P1NP (r = −0.288, *p* = 0.001), CTX-1 (r = +0.205, *p* = 0.022), NRP1 (r = −0.331, *p* < 0.001), PLXNA1 (r = +0.228, *p* = 0.010), P1NP/CTX-1 (r = −0.368, *p* < 0.001), NRP1/PLXNA1 (r = −0.501, *p* < 0.001), and SEMA3A/NRP1 (r = +0.249, *p* = 0.005). CV was significantly correlated with P1NP (r = −0.272, *p* = 0.002), CTX-1 (r = +0.314, *p* < 0.001), NRP1 (r = −0.214, *p* = 0.016), PLXNA1 (r = +0.403, *p* < 0.001), P1NP/CTX-1 (r = −0.415, *p* < 0.001), NRP1/PLXNA1 (r = −0.490, *p* < 0.001), and SEMA3A/NRP1 (r = +0.205, *p* = 0.021). SD was significantly correlated with P1NP (r = −0.281, *p* = 0.002), CTX-1 (r = +0.287, *p* = 0.001), NRP1 (r = −0.210, *p* = 0.018), PLXNA1 (r = +0.333, *p* < 0.001), P1NP/CTX-1 (r = −0.395, *p* < 0.001), NRP1/PLXNA1 (r = −0.451, *p* < 0.001), and SEMA3A/NRP1 (r = +0.190, *p* = 0.033). Mean glucose and postprandial increment showed no significant correlation with any marker or index (all *p* > 0.05). SEMA3A showed no significant correlation with any glycemic variability parameter (all *p* > 0.05). L1–L4, femoral neck, and total femur T-scores showed no significant correlation with any glycemic variability parameter (all *p* > 0.05). The relationship between Δ glucose and the P1NP/CTX-1 and NRP1/PLXNA1 ratios is shown in Figure 2.

Table 3 presents the factors independently associated with bone turnover indices and biomarkers assessed by multiple regression analysis. The model for the P1NP/CTX-1 ratio was significant (R^2^ = 0.287, *p* < 0.001); CV (per 1-percentage-point increase) was independently associated with the ratio (B = −0.00174, *p* = 0.017), and age (B = −0.0017, *p* = 0.041), BMI (B = −0.0069, *p* = 0.024), vitamin D (B = +0.00096, *p* = 0.020), PTH (B = +0.00074, *p* = 0.005), and insulin use (B = +0.0210, *p* = 0.033) were also significantly associated in the same model. The model for P1NP was significant (R^2^ = 0.213, *p* = 0.001); age (B = −1.234, *p* = 0.009) and BMI (B = −3.857, *p* = 0.025) were independently associated, while none of the glycemic variability parameters was significant. The model for CTX-1 was not significant (R^2^ = 0.050, *p* = 0.727).

Table 4 examines the factors independently associated with circulating Sema3A-related biomarkers and related indices. The model for the NRP1/PLXNA1 ratio was significant (R^2^ = 0.240, *p* < 0.001); Δ glucose was independently associated with the ratio (B = −0.00023, *p* = 0.005). The model for NRP1 was significant (R^2^ = 0.155, *p* = 0.018); Δ glucose was independently associated with NRP1 (B = −0.205, *p* = 0.013). The model for PLXNA1 was not significant R^2^ = 0.058, *p* = 0.623); no variable was significantly associated with PLXNA1. The model for the SEMA3A/NRP1 ratio was significant (R^2^ = 0.226, *p* < 0.001); mean glucose (B = −0.00022, *p* = 0.021) and Δ glucose (B = +0.00035, *p* < 0.001) were independently associated with the ratio. The quartile-based relationship between bone turnover indices and glycemic variability is shown in Supplementary Figure S1, and detailed values for Δ glucose quartiles are presented in Supplementary Table S3.

## 4. Discussion

This study evaluated the relationships between glycemic variability and bone turnover markers and circulating Sema3A-related biomarkers in postmenopausal women with T2DM. No significant differences were found among HbA1c-based groups in bone turnover markers, Sema3A pathway components, or DXA results. In contrast, SD, CV, and Δ glucose were associated with several biomarkers and ratios in unadjusted analyses. In multivariate models, CV was independently associated with the P1NP/CTX-1 ratio, while Δ glucose was independently associated with NRP1 and the NRP1/PLXNA1 ratio. The SEMA3A/NRP1 ratio was also positively associated with Δ glucose and negatively associated with mean glucose. These findings suggest that short-term glycemic variability—predominantly reflecting within-day glucose fluctuations and not captured by average glycemia alone—was associated with the balance of bone turnover and with the relative levels of circulating Sema3A receptor components.

### 4.1. Limitations of HbA1c-Based Classification in Distinguishing Bone Metabolism

HbA1c is one of the principal indicators used to assess long-term average glycemic control but does not reflect within-day glycemic variability. The literature on the relationship between HbA1c and bone turnover is likewise inconsistent. Farr and colleagues [19] and Wang and colleagues [20] reported negative associations, whereas Kanazawa and colleagues [21] found no significant association. Miazgowski and colleagues similarly showed that improvement in glycemic control was not accompanied by a parallel improvement in bone turnover markers [22]. The absence of differences among HbA1c groups in bone turnover markers and Sema3A-related biomarkers in our study supports the notion that average glycemia alone may be insufficient to explain biochemical variability in diabetic bone disease. This was corroborated by a sensitivity analysis in which HbA1c was entered as a continuous covariate alongside the primary glycemic-variability metrics: HbA1c itself was not significantly associated with any outcome, and the coefficients for CV and Δ glucose remained materially unchanged (Supplementary Table S6f), supporting the conclusion that these associations are not attributable to longer-term average glycemic exposure.

### 4.2. Reflections of Glycemic Variability Indices on Bone Turnover Biomarkers

In unadjusted correlation analyses, P1NP and the P1NP/CTX-1 ratio decreased while CTX-1 increased as SD, CV, and Δ glucose increased. This pattern suggests that the balance between bone formation and resorption may shift in a relatively unfavorable direction with increasing glycemic variability. Our findings are broadly consistent with those of Huang and colleagues, who linked high glycemic variability with osteoporosis [10], and Starup-Linde and colleagues, who reported that glucose variability was associated with low bone turnover independently of mean glycemia [12]. Possible biological explanations for this relationship include increased oxidative stress from recurrent glucose fluctuations, suppression of osteoblast differentiation, and promotion of osteoclastogenesis via the RANKL/OPG balance [5].

However, the multivariate results warrant more cautious interpretation. In the model for P1NP, the glycemic variability indices did not retain independent associations, and age and BMI emerged as the significant variables; the model for CTX-1 was not significant overall. In contrast, CV retained its independent negative association with the P1NP/CTX-1 ratio. This suggests that the relationship between glycemic variability and bone turnover may become apparent primarily through the relative balance between these two markers rather than through P1NP or CTX-1 individually.

SD, CV, and Δ glucose represent related but distinct dimensions of glycemic variability: SD reflects absolute variability, CV reflects variability relative to mean glucose, and Δ glucose reflects the daily amplitude between peak and trough values. The independent associations of CV with P1NP/CTX-1 and of Δ glucose with NRP1 and NRP1/PLXNA1 suggest that these indices may capture different biological aspects of glycemic variability. However, this does not establish the clinical superiority of one index over another.

### 4.3. The Link Between Circulating Sema3A-Related Biomarkers and Glycemic Variability

One of the distinctive aspects of this study is the joint evaluation of glycemic variability and circulating Sema3A pathway components. Sema3A has been shown to support osteoblast differentiation and suppress osteoclast formation via the NRP1–PLXNA1 receptor complex [13]. Exposure to hyperglycemia and advanced glycation end products may impair Sema3A-related osteogenic processes [14], while Sema3A administration has been reported to support osteogenic differentiation in diabetic animal models [23]. Genetic variation in the SEMA3A pathway has also been associated with BMD and osteoporosis risk, further supporting the pathway’s relevance to bone biology [24].

In our study, SEMA3A levels were not associated with any glycemic parameter, whereas Δ glucose showed independent negative associations with NRP1 and the NRP1/PLXNA1 ratio. This dissociation suggests that glycemic variability may be associated with the relative levels of receptor components rather than with circulating ligand levels. However, the extent to which serum NRP1 and PLXNA1 levels reflect membrane expression on bone cells or intracellular signaling activity remains unknown. Our findings should therefore not be interpreted as direct evidence of receptor complex expression or Sema3A signaling activity. The NRP1/PLXNA1 ratio is an exploratory index defined a priori for this study to examine the relative balance between receptor components. It is possible that the negative association between Δ glucose and this ratio reflects both a decrease in NRP1 and a relative change in PLXNA1; however, the non-significance of the PLXNA1 model suggests that the adjusted relationship may be driven predominantly by the NRP1 component. The SEMA3A/NRP1 ratio also showed an independent positive association with Δ glucose (B = 0.00035; *p* < 0.001) and a negative association with mean glucose (B = −0.00022; *p* = 0.021). Given that SEMA3A itself was not associated with glycemic indices and that NRP1 was negatively associated with Δ glucose, the rise in this ratio is plausibly explained, at least in part, by the decrease in NRP1 in the denominator. The SEMA3A/NRP1 ratio should therefore be regarded as an exploratory index reflecting the relative balance between the ligand and its binding co-receptor, rather than an independent measure of signaling activity. Because the P1NP/CTX-1 and NRP1/PLXNA1 ratios showed right-skewed distributions, we verified that the CV and Δ glucose associations reported above were not artifacts of ratio scaling by re-modeling each ratio in log-transformed form [ln(numerator) − ln(denominator)]; all three associations remained significant, and were somewhat stronger, in the log-transformed models (Supplementary Table S6e).

### 4.4. Absence of Differences in DXA Values and the Hypothesis-Generating Potential of Circulating Biomarkers

No differences were observed among HbA1c groups in lumbar spine, femoral neck, or total femur T-scores, and glycemic variability indices were not associated with regional T-scores. This finding was consistent when bone mineral density was examined in absolute terms (g/cm^2^) rather than as T-scores (Supplementary Table S8). Fracture risk can be elevated in T2DM despite normal or increased BMD [4]. This discrepancy has been attributed to DXA’s inability to directly assess components of bone quality, such as microarchitecture, collagen structure, and cortical porosity [5]. The fact that bone turnover markers reflect current biochemical activity, whereas DXA reflects cumulative mineral content accrued over a longer period, may also partly explain the discordance between these findings. However, the cross-sectional design does not establish that biomarker changes precede BMD loss. TBS could not be measured in our study due to equipment limitations. TBS is a validated measure known to contribute to fracture risk prediction independently of BMD [25]; whether our exploratory circulating biomarkers could provide comparable complementary information is unknown and cannot be inferred from the present cross-sectional data. The decrease in P1NP, increase in CTX-1, and decrease in NRP1/PLXNA1 observed in the high-variability group may represent a hypothesis-generating signal of possible bone quality or signaling impairment despite normal DXA values, warranting evaluation in longitudinal studies.

### 4.5. Independent Clinical Determinants of Bone Metabolism: PTH and Insulin

In the P1NP/CTX-1 model, age and BMI were negatively associated, while vitamin D, PTH, and insulin use were positively associated, in addition to CV. These findings indicate that bone turnover is shaped not only by glycemic variability but also by age, body composition, hormonal status, and treatment characteristics. Intermittent PTH secretion is known to be potentially anabolic, whereas sustained elevation may be catabolic [26]; when secondary hyperparathyroidism due to vitamin D insufficiency and the low-turnover state associated with T2DM itself, which suppresses PTH [27], coexist in postmenopausal T2DM, these opposing tendencies may explain why PTH emerged as a factor strongly and independently associated with the ratio. However, a single PTH measurement does not capture the hormone’s net anabolic or catabolic effect on bone. Similarly, although the positive association of insulin use is consistent with experimental data demonstrating an anabolic effect of insulin on osteoblasts [18], it cannot be fully separated from indication-related confounding. These findings should be regarded not as causal conclusions but as associations warranting further investigation.

### 4.6. Δ Glucose Showed the Strongest Association in This Sample

After adjustment for age, BMI, diabetes duration, vitamin D, PTH, and insulin use, Δ glucose was the only glycemic variability parameter independently associated with the NRP1/PLXNA1 ratio (B = −0.00023; *p* = 0.005) and with NRP1 (B = −0.205; *p* = 0.013); it also showed the strongest correlations and the most pronounced graded pattern across quartiles. Mean glucose, SD, CV, and Δ glucose were themselves moderately-to-strongly inter-correlated (r = 0.31–0.88; Table 2b), and when CV and Δ glucose were modeled individually against mean glucose (excluding the competing metric), both were broadly significant across the seven outcome models; once entered together in the mutually adjusted models reported in Table 3 and Table 4, only one metric per outcome retained significance—the expected pattern given this shared variance, rather than a data inconsistency (Supplementary Table S6c). This is consistent with Δ glucose being a clinically practical index that reflects the daily peak-to-trough range and can be calculated simply as the difference between the highest and lowest values of the day [28]. Although a CV threshold of 36% is often cited in consensus recommendations [29], this threshold is largely derived from CGM data; in home-based measurements, Δ glucose is considered more readily calculable. Quartile analysis in our sample suggested a graded relationship: a Δ glucose <100 mg/dL was characterized by relatively preserved indices (P1NP/CTX-1 ~0.10; NRP1/PLXNA1 ~0.12), whereas a Δ glucose >150 mg/dL was characterized by markedly lower indices (~0.07; ~0.08). However, these are sample-derived, post hoc categories that were not defined by ROC analysis or validated against a clinical outcome, and they should not be interpreted as clinical thresholds; they require independent, outcome-based validation before any such use.

### 4.7. Strengths of the Study

Our study has several distinctive strengths. Unlike studies relying on CGM, we evaluated the clinical value of frequent home-based capillary monitoring as a potentially accessible complementary approach. Bone metabolism was assessed not only through classic markers such as P1NP and CTX-1 but also through circulating Sema3A-related components—NRP1, PLXNA1, and SEMA3A—using an exploratory index intended to capture the relative balance between receptor components rather than a validated indicator of receptor signaling activity.

In addition, important confounders (age, BMI, diabetes duration, vitamin D, PTH, insulin use) were controlled for through multivariate regression analyses, and VIF values below 5 supported the reliability of the models with respect to collinearity. The final analytic sample (*n* = 126) exceeded the pre-specified target (*n* = 109); however, missingness was handled through complete-case exclusion of 13 participants rather than imputation, and as detailed in Section 2.2 and Supplementary Table S7, excluded patients differed from included patients in comorbidity burden (hypertension, coronary artery disease), so the possibility of selection bias cannot be fully excluded. Studying a homogeneous population (postmenopausal women with T2DM) reduced heterogeneity related to sex and menopausal status, but this restriction does not eliminate confounding and may limit generalizability to other populations.

### 4.8. Limitations of the Study

Beyond the within-day fluctuations captured by our capillary glucose protocol, bone turnover markers and glucose metabolism are also embedded in broader circadian and behavioral rhythms (for example, diurnal variation in bone resorption markers, meal timing, and sleep–wake cycles) that were not separately modeled in this study. Distinguishing short-term glycemic variability, as measured here, from this broader chronobiological context is a useful direction for future research on the bone-muscle unit in metabolic disease [30].

Our study has several limitations. The cross-sectional design precludes determination of the causal direction between glycemic variability and bone turnover; this can only be clarified through longitudinal studies. Glycemic variability data were derived from five days of home-based capillary measurements at fixed time points rather than continuously; although this offers clinical feasibility, it involves fewer measurement points than CGM, may not capture longer-term variability such as weekday–weekend differences or seasonal effects, and may miss the true postprandial glucose peak or nocturnal glucose trough occurring between sampling times, potentially leading to underestimation of Δ glucose in some participants. The homogeneity of the population (postmenopausal women with T2DM only) strengthens internal validity but limits generalizability to men, premenopausal women, and individuals with type 1 diabetes. Data on menopause duration, smoking, physical activity, and fracture history were not systematically collected and could not be included as covariates; this should be considered when interpreting the independence of the reported associations. In addition, the study did not include a non-diabetic comparison group, so the discussion of ‘diabetic bone disease’ mechanisms reflects associations observed within a T2DM population rather than a direct comparison with non-diabetic bone metabolism.

The extent to which serum SEMA3A, NRP1, and PLXNA1 levels reflect local expression in bone tissue is uncertain; bone biopsy or histomorphometric confirmation was not performed, and this relationship was inferred indirectly. TBS could not be measured due to equipment limitations, precluding direct assessment of bone microarchitecture. Finally, the single-center design and moderate sample size may have limited the power of some subgroup analyses, and the insulin-related finding cannot be fully separated from indication bias. Our findings require validation in multicenter studies with larger samples.

## 5. Conclusions

This study found that short-term glycemic variability—particularly Δ glucose—was associated with bone turnover measures and exploratory Sema3A-related biomarker ratios independently of average glycemic control and bone mineral density. HbA1c-based glycemic control classification did not distinguish bone turnover markers or DXA parameters, whereas variability indices such as SD, CV, and Δ glucose showed consistent associations with the bone formation–resorption balance (P1NP/CTX-1) and the Sema3A receptor complex (NRP1/PLXNA1, SEMA3A/NRP1). In adjusted multivariate analyses, CV was independently associated with the P1NP/CTX-1 ratio, while Δ glucose was repeatedly and independently associated with Sema3A-related indices (NRP1, NRP1/PLXNA1, SEMA3A/NRP1); both associations persisted after adjustment for clinically important confounders such as age, BMI, diabetes duration, vitamin D, PTH, and insulin use.

These findings suggest that short-term (predominantly within-day) glucose variability, not captured by average glycemic control alone, may warrant consideration in future prospective studies of bone health in postmenopausal women with T2DM; whether the home-based capillary monitoring approach used here could serve as a practical complementary tool alongside CGM in such studies remains to be evaluated. The NRP1/PLXNA1 and SEMA3A/NRP1 ratios should be regarded as hypothesis-generating candidate biomarkers for diabetic bone disease rather than validated early-warning tools; these findings require validation in longitudinal, multicenter studies with larger samples before they can be interpreted causally or considered for clinical translation.

## Figures and Tables

**Figure 1:**
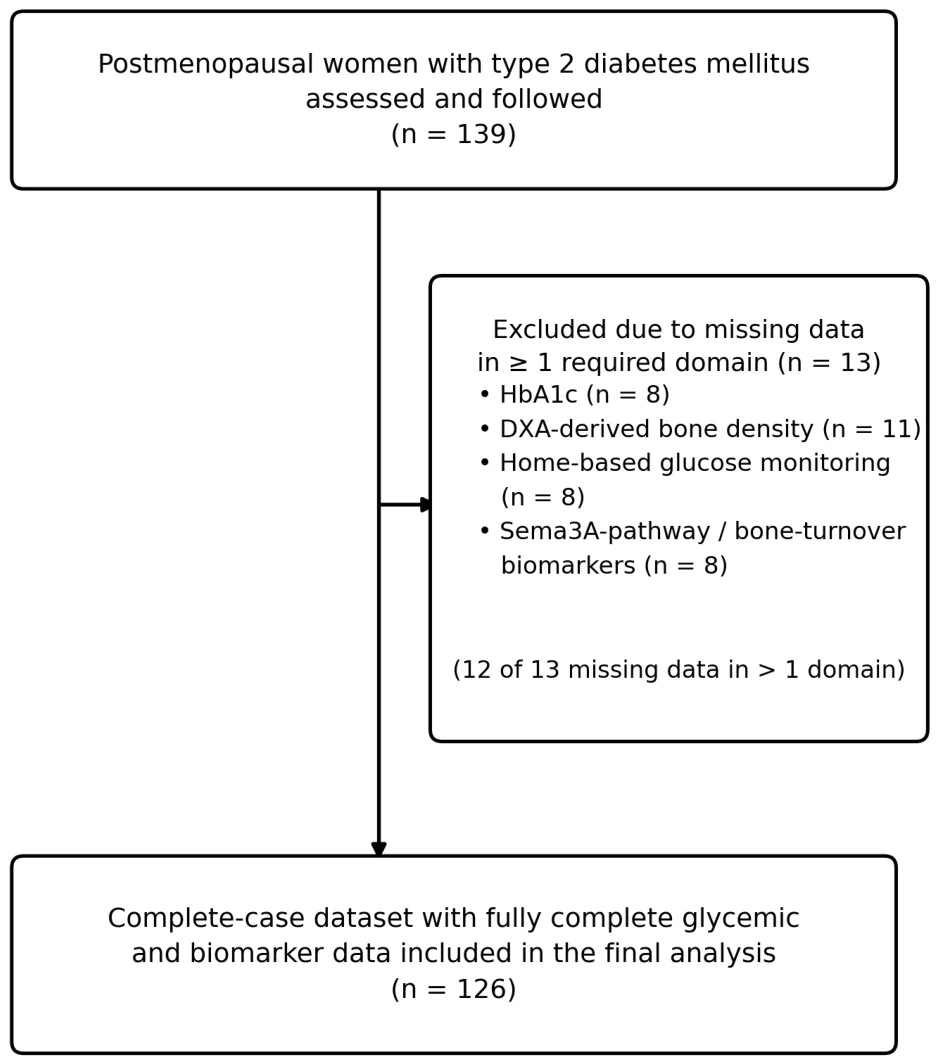
Participant flow diagram. Flowchart summarizing enrollment, reasons for exclusion, and the final complete-case analytic sample. Of 139 postmenopausal women with type 2 diabetes mellitus initially followed, 13 were excluded because of missing data in at least one required domain (HbA1c, DXA-derived bone density, home-based capillary glucose monitoring, or Sema3A-pathway/bone turnover biomarkers), leaving 126 women in the final analysis. DXA: dual-energy X-ray absorptiometry.

**Figure 2:**
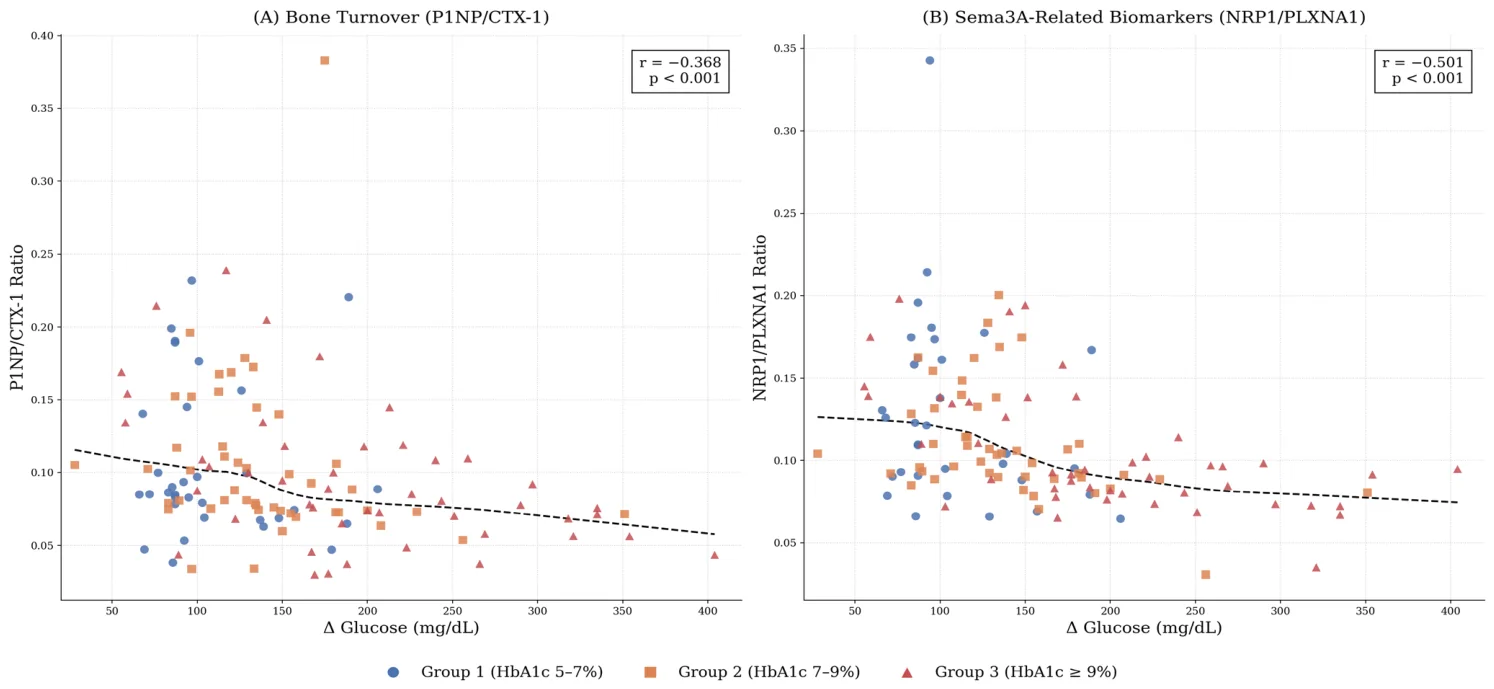
Relationship between Δ glucose and bone turnover/Sema3A-related biomarkers. *(***A**) Scatter plot showing the correlation between Δ glucose and the P1NP/CTX-1 ratio, a bone turnover formation/resorption index (Spearman r = −0.368, *p* < 0.001). (**B**) Scatter plot showing the correlation between Δ glucose and the NRP1/PLXNA1 ratio, reflecting the relative balance of circulating Sema3A-related receptor components (Spearman r = −0.501, *p* < 0.001). Because Spearman correlation is rank-based, dashed lines represent a LOWESS (locally weighted) smoother shown for visualization purposes only and do not imply a linear regression model. Points are colored according to glycemic control group (Group 1: HbA1c 5–7%; Group 2: HbA1c 7–9%; Group 3: HbA1c ≥ 9%) for visualization purposes only; group membership was not used to test statistical independence. P1NP: procollagen type 1 N-terminal propeptide; CTX-1: C-terminal telopeptide type 1; NRP1: Neuropilin-1; PLXNA1: Plexin-A1.

**Table 1:** Demographic, Clinical, and Bone Turnover Characteristics of Patients According to Glycemic Control Status. Group 1: HbA1c 5–7% [31–53 mmol/mol] (*n* = 33)|Group 2: HbA1c 7–9% [53–75 mmol/mol] (*n* = 47)|Group 3: HbA1c ≥9.0% [≥75 mmol/mol] (*n* = 46).

Variable	All Patients (*n* = 126)	Group 1 (*n* = 33)	Group 2 (*n* = 47)	Group 3 (*n* = 46)	*p* Value
**Demographic characteristics**					
Age (years)	64.00 [59.00–67.75]	64.00 [61.00–67.00]	64.00 [58.50–67.00]	64.00 [58.00–68.00]	0.661
DM duration (years)	13.00 [10.00–15.00]	11.00 [8.00–14.00]	13.00 [10.00–15.50]	15.00 [11.00–15.00]	**0.011** 1–3: *
BMI (kg/m^2^)	28.70 [28.00–29.20]	28.50 [27.90–29.00]	28.80 [28.10–29.70]	28.90 [28.23–29.20]	0.240
**Glycemic parameters**					
HbA1c % (mmol/mol)	8.10 [7.01–9.60] 65 [53–81]	6.50 [6.20–6.80] 48 [44–51]	7.80 [7.40–8.35] 62 [57–68]	10.50 [9.33–11.57] 91 [78–103]	**<0.001** 1–2: * 1–3: * 2–3: *
FPG (mg/dL)	158.50 [126.00–204.75]	124.00 [105.00–140.00]	162.00 [128.50–200.00]	202.50 [155.25–282.75]	**<0.001** 1–2: * 1–3: * 2–3: *
**Lipid profile**					
Total cholesterol (mg/dL)	190.00 [160.50–213.25]	189.00 [164.00–204.00]	179.00 [148.50–205.50]	200.50 [164.00–226.75]	0.161
LDL (mg/dL)	101.88 ± 31.44	103.30 ± 30.10	98.87 ± 30.36	103.93 ± 33.83	0.710
HDL (mg/dL)	49.00 [41.00–57.00]	48.00 [40.00–54.00]	49.00 [42.00–60.00]	50.00 [42.00–56.50]	0.875
Triglycerides (mg/dL)	159.50 [123.50–216.75]	163.00 [109.00–216.00]	140.00 [112.00–179.00]	173.50 [136.25–230.75]	0.093
**Bone turnover markers and SEMA3A signal**					
P1NP (ng/mL)	39.79 [33.59–63.23]	42.98 [36.20–72.44]	38.40 [34.54–55.45]	37.77 [31.86–61.31]	0.339
CTX-1 (ng/mL)	470.59 [415.16–518.61]	457.64 [413.91–512.45]	461.73 [414.95–511.77]	476.18 [424.84–546.86]	0.555
NRP1 (ng/mL)	81.69 [72.10–115.80]	87.61 [77.47–134.66]	81.21 [71.89–99.99]	80.16 [70.62–102.14]	0.167
PLXNA1 (ng/mL)	825.86 [764.14–910.71]	853.29 [789.57–900.86]	793.71 [754.50–866.86]	833.21 [781.43–943.43]	0.254
SEMA3A (ng/mL)	9.67 [7.48–10.84]	9.78 [7.88–12.01]	9.52 [7.75–10.49]	9.57 [6.71–10.70]	0.518
**Bone turnover and SEMA3A indices**					
P1NP/CTX-1 (coupling)	0.09 [0.07–0.12]	0.09 [0.07–0.14]	0.09 [0.07–0.12]	0.08 [0.07–0.12]	0.571
NRP1/PLXNA1	0.10 [0.09–0.14]	0.11 [0.09–0.16]	0.10 [0.09–0.13]	0.09 [0.08–0.13]	0.144
SEMA3A/NRP1	0.12 [0.07–0.14]	0.12 [0.06–0.14]	0.12 [0.10–0.14]	0.13 [0.07–0.14]	0.928
**Bone density**					
L1-L4 T-score	−1.00 [−2.18–0.10]	−0.80 [−1.80–0.10]	−1.40 [−2.30–0.06]	−1.10 [−2.10–0.20]	0.455
Femoral neck T-score	−1.10 [−1.80–0.43]	−1.10 [−1.80–0.40]	−1.20 [−1.65–0.50]	−1.00 [−1.80–0.30]	0.988
Total femur T-score	−1.00 [−1.60–0.40]	−1.20 [−1.50–0.60]	−1.00 [−1.70–0.45]	−1.00 [−1.50–0.23]	0.799

Data are presented as median [interquartile range] or mean ± SD depending on distribution; normality was assessed using the Shapiro–Wilk test. Groups were compared using one-way ANOVA or the Kruskal–Wallis test, with Bonferroni-corrected post hoc comparisons performed when overall tests were significant (indicated by *); *p* < 0.05 was considered statistically significant. No significant differences were found between groups for any bone turnover marker, index, or bone density parameter (all *p* > 0.05). DM: diabetes mellitus; BMI: body mass index; HbA1c: hemoglobin A1c; FPG: fasting plasma glucose; LDL: low-density lipoprotein; HDL: high-density lipoprotein; P1NP: procollagen type 1 N-terminal propeptide; CTX-1: C-terminal telopeptide type 1; NRP1: Neuropilin-1; PLXNA1: Plexin-A1; SEMA3A: Semaphorin 3A; P1NP/CTX-1: bone turnover formation/resorption coupling ratio; NRP1/PLXNA1 and SEMA3A/NRP1: exploratory Sema3A-related biomarker ratios. All biomarkers were expressed in ng/mL before calculation of the ratios; P1NP and CTX-1 were expressed in the same unit (ng/mL), so the P1NP/CTX-1 ratio is dimensionless. Bold values indicate statistically significant correlations (*p* < 0.05).

**Table 2:** (**a**) Correlation analysis between glycemic variability parameters and bone turnover markers and bone density (Spearman, *n* = 126). (**b**) Spearman correlations among glycemic variability indices (*n* = 126).

(**a**)					
	**Mean Glucose**	**SD**	**CV**	**Δ Glucose**	**Postprandial Increment**
P1NP (ng/mL)	r = −0.141	r = −0.281	r = −0.272	r = −0.288	r = −0.173
	*p* = 0.114	***p*** ** = 0.002**	***p*** ** = 0.002**	***p*** ** = 0.001**	*p* = 0.052
CTX-1 (ng/mL)	r = +0.143	r = +0.287	r = +0.314	r = +0.205	r = +0.126
	*p* = 0.109	***p*** ** = 0.001**	***p*** ** < 0.001**	***p*** ** = 0.022**	*p* = 0.158
NRP1 (ng/mL)	r = −0.052	r = −0.210	r = −0.214	r = −0.331	r = −0.072
	*p* = 0.562	***p*** ** = 0.018**	***p*** ** = 0.016**	***p*** ** < 0.001**	*p* = 0.426
PLXNA1 (ng/mL)	r = +0.104	r = +0.333	r = +0.403	r = +0.228	r = +0.167
	*p* = 0.247	***p*** ** < 0.001**	***p*** ** < 0.001**	***p*** ** = 0.010**	*p* = 0.062
SEMA3A (ng/mL)	r = −0.086	r = +0.020	r = +0.028	r = +0.016	r = −0.081
	*p* = 0.340	*p* = 0.827	*p* = 0.759	*p* = 0.859	*p* = 0.365
P1NP/CTX-1 (coupling)	r = −0.135	r = −0.395	r = −0.415	r = −0.368	r = −0.171
	*p* = 0.133	***p*** ** < 0.001**	***p*** ** < 0.001**	***p*** ** < 0.001**	*p* = 0.055
NRP1/PLXNA1	r = −0.117	r = −0.451	r = −0.490	r = −0.501	r = −0.144
	*p* = 0.193	***p*** ** < 0.001**	***p*** ** < 0.001**	***p*** ** < 0.001**	*p* = 0.107
SEMA3A/NRP1	r = −0.025	r = +0.190	r = +0.205	r = +0.249	r = −0.008
	*p* = 0.784	***p*** ** = 0.033**	***p*** ** = 0.021**	***p*** ** = 0.005**	*p* = 0.932
L1-L4 T-score	r = −0.056	r = −0.105	r = −0.058	r = −0.090	r = −0.098
	*p* = 0.532	*p* = 0.241	*p* = 0.517	*p* = 0.315	*p* = 0.274
Femoral neck T-score	r = −0.114	r = −0.121	r = −0.130	r = −0.069	r = −0.115
	*p* = 0.202	*p* = 0.177	*p* = 0.146	*p* = 0.442	*p* = 0.198
Total femur T-score	r = −0.017	r = +0.042	r = +0.002	r = +0.037	r = −0.105
	*p* = 0.850	*p* = 0.640	*p* = 0.980	*p* = 0.683	*p* = 0.242
(**b**)					
	**Mean Glucose**	**SD**	**CV (%)**	**Δ Glucose**	**Postprandial Increment**
Mean glucose	1	0.52	0.31	0.5	0.39
SD	0.52	1	0.88	0.86	0.6
CV (%)	0.31	0.88	1	0.79	0.6
Δ glucose	0.5	0.86	0.79	1	0.52
Postprandial increment	0.39	0.6	0.6	0.52	1

Spearman correlation coefficients (r) and *p*-values are presented. Bold values indicate statistically significant correlations (*p* < 0.05). Raw (uncorrected) *p*-values are shown; correlation analyses are exploratory. Given the number of comparisons performed (55 correlations), a Benjamini–Hochberg false discovery rate (FDR) correction was also applied. Of the 21 correlations significant at raw *p* < 0.05, 16 remained significant at FDR q < 0.05, while five (NRP1–CV, NRP1–SD, SEMA3A/NRP1–CV, CTX-1–Δ glucose, SEMA3A/NRP1–SD) did not survive correction and should be interpreted with caution. Full q-values are provided in Supplementary Table S4. SD: standard deviation; CV: coefficient of variation; P1NP: procollagen type 1 N-terminal propeptide; CTX-1: C-terminal telopeptide type 1; NRP1: Neuropilin-1; PLXNA1: Plexin-A1; SEMA3A: Semaphorin 3A; P1NP/CTX-1: bone turnover formation/resorption coupling ratio; NRP1/PLXNA1 and SEMA3A/NRP1: exploratory Sema3A-related biomarker ratios. All biomarkers were expressed in ng/mL before calculating the ratios; P1NP and CTX-1 were expressed in the same unit (ng/mL), so the P1NP/CTX-1 ratio is dimensionless; Spearman correlation coefficients among the five glycemic variability indices calculated from the five-day home capillary monitoring protocol. SD, CV, and Δ glucose are highly inter-correlated (r = 0.79–0.88), consistent with their shared derivation from the same capillary glucose measurements; this pattern underlies the VIF values reported in Supplementary Table S6a and motivated the single-metric sensitivity models (CV-only and Δ glucose-only, Supplementary Table S6c) summarized in Section 4.6. SD: standard deviation; CV: coefficient of variation.

**Table 3:** Factors independently associated with bone turnover indices and biomarkers: multivariate linear regression.

Independent Variable	B	Standard Error	95% CI	*p* Value
**Model 1: P1NP/CTX-1 (coupling)—R^2^ = 0.287; Adjusted R^2^ = 0.232; *p* < 0.001**				
Age	**−0.00168**	0.00081	[−0.00329; −0.00007]	**0.041**
BMI	**−0.00685**	0.00298	[−0.01276; −0.00094]	**0.024**
DM duration	+0.00087	0.00127	[−0.00165; +0.00338]	0.495
Vitamin D	**+0.00096**	0.00041	[+0.00015; +0.00177]	**0.020**
PTH	**+0.00074**	0.00026	[+0.00024; +0.00125]	**0.004**
Insulin use	**+0.02100**	0.00973	[+0.00172; +0.04028]	**0.033**
Mean glucose	−0.00000	0.00010	[−0.00020; +0.00020]	0.994
CV (%)	**−0.00174**	0.00072	[−0.00316; −0.00032]	**0.017**
Δ Glucose	−0.00008	0.00010	[−0.00028; +0.00011]	0.382
**Model 2: P1NP—R^2^ = 0.213; Adjusted R^2^ = 0.152; *p* = 0.001**				
Age	**−1.23393**	0.46122	[−2.14752; −0.32033]	**0.009**
BMI	**−3.85732**	1.69180	[−7.20845; −0.50619]	**0.025**
DM duration	+0.97174	0.71984	[−0.45412; +2.39760]	0.180
Vitamin D	+0.15652	0.23199	[−0.30300; +0.61605]	0.501
PTH	+0.27435	0.14554	[−0.01393; +0.56263]	0.062
Insulin use	+8.85376	5.52055	[−2.08140; +19.78891]	0.112
Mean glucose	−0.01260	0.05644	[−0.12440; +0.09919]	0.824
CV (%)	−0.48686	0.40669	[−1.29244; +0.31872]	0.234
Δ Glucose	−0.06136	0.05494	[−0.17018; +0.04747]	0.266
**Model 3: CTX-1—R^2^ = 0.050; Adjusted R^2^ = −0.024; *p* = 0.727**				
Age	−2.60711	3.04882	[−8.64624; +3.43201]	0.394
BMI	−8.01647	11.18328	[−30.16841; +14.13546]	0.475
DM duration	+6.04112	4.75833	[−3.38421; +15.46646]	0.207
Vitamin D	−1.85768	1.53352	[−4.89530; +1.17993]	0.228
PTH	−0.18028	0.96204	[−2.08588; +1.72533]	0.852
Insulin use	−3.07456	36.49246	[−75.35909; +69.20997]	0.933
Mean glucose	−0.24755	0.37307	[−0.98653; +0.49143]	0.508
CV (%)	+3.99888	2.68836	[−1.32624; +9.32401]	0.140
Δ Glucose	−0.25750	0.36317	[−0.97687; +0.46188]	0.480

B: unstandardized regression coefficient; SE: standard error; 95% CI: 95% confidence interval; R^2^: coefficient of determination. Bold values indicate a statistically significant result (*p* < 0.05). P1NP: procollagen type 1 N-terminal propeptide; CTX-1: C-terminal telopeptide type 1; P1NP/CTX-1: bone turnover formation/resorption coupling ratio; PTH: parathyroid hormone; BMI: body mass index; DM: diabetes mellitus; CV: coefficient of variation. CV coefficients represent the estimated change per 1-percentage-point increase in CV. Variance inflation factors and tolerance values for all covariates (identical across models) are reported in Supplementary Table S6.

**Table 4:** Factors independently associated with circulating Sema3A-related biomarkers (NRP1, PLXNA1) and related indices: multivariate linear regression.

Independent Variable	B	Standard Error	95% CI	*p* Value
**Model 1: NRP1/PLXNA1—R^2^ = 0.240; Adjusted R^2^ = 0.180; *p* < 0.001**				
Age	−0.00008	0.00068	[−0.00143; +0.00127]	0.911
BMI	−0.00233	0.00250	[−0.00729; +0.00263]	0.354
DM duration	+0.00099	0.00106	[−0.00112; +0.00310]	0.355
Vitamin D	+0.00038	0.00034	[−0.00030; +0.00106]	0.275
PTH	+0.00031	0.00022	[−0.00012; +0.00073]	0.157
Insulin use	+0.00479	0.00817	[−0.01138; +0.02097]	0.558
Mean glucose	+0.00006	0.00008	[−0.00010; +0.00023]	0.467
CV (%)	−0.00064	0.00060	[−0.00184; +0.00055]	0.286
Δ Glucose	**−0.00023**	0.00008	[−0.00040; −0.00007]	**0.005**
**Model 2: SEMA3A/NRP1—R^2^ = 0.226; Adjusted R^2^ = 0.165; *p* < 0.001**				
Age	+0.00091	0.00075	[−0.00059; +0.00240]	0.233
BMI	+0.00325	0.00277	[−0.00223; +0.00874]	0.242
DM duration	−0.00115	0.00118	[−0.00349; +0.00118]	0.330
Vitamin D	+0.00006	0.00038	[−0.00069; +0.00082]	0.868
PTH	−0.00046	0.00024	[−0.00094; +0.00001]	0.054
Insulin use	−0.01174	0.00904	[−0.02964; +0.00616]	0.197
Mean glucose	**−0.00022**	0.00009	[−0.00040; −0.00003]	**0.021**
CV (%)	−0.00045	0.00067	[−0.00176; +0.00087]	0.504
Δ Glucose	**+0.00035**	0.00009	[+0.00017; +0.00053]	**<0.001**
**Model 3: PLXNA1—R^2^ = 0.058; Adjusted R^2^ = −0.015; *p* = 0.623**				
Age	−4.26037	5.32446	[−14.80709; +6.28636]	0.425
BMI	+6.99727	19.53047	[−31.68883; +45.68337]	0.721
DM duration	−0.64066	8.30994	[−17.10106; +15.81973]	0.939
Vitamin D	−0.87183	2.67814	[−6.17672; +4.43305]	0.745
PTH	+0.23715	1.68010	[−3.09080; +3.56510]	0.888
Insulin use	+10.08668	63.73036	[−116.15090; +136.32425]	0.875
Mean glucose	−0.34071	0.65153	[−1.63126; +0.94984]	0.602
CV (%)	+5.29447	4.69495	[−4.00531; +14.59426]	0.262
Δ Glucose	+0.40119	0.63425	[−0.85514; +1.65751]	0.528
**Model 4: NRP1—R^2^ = 0.155; Adjusted R^2^ = 0.089; *p* = 0.018**				
Age	−0.86899	0.68249	[−2.22087; +0.48290]	0.205
BMI	−2.97389	2.50342	[−7.93268; +1.98491]	0.237
DM duration	+1.17957	1.06517	[−0.93032; +3.28947]	0.270
Vitamin D	−0.04314	0.34328	[−0.72312; +0.63684]	0.900
PTH	+0.23938	0.21536	[−0.18720; +0.66596]	0.269
Insulin use	+8.21745	8.16897	[−7.96371; +24.39861]	0.317
Mean glucose	+0.03401	0.08351	[−0.13141; +0.19943]	0.685
CV (%)	−0.04482	0.60180	[−1.23687; +1.14722]	0.941
Δ Glucose	**−0.20490**	0.08130	[−0.36594; −0.04387]	**0.013**

B: unstandardized regression coefficient; SE: standard error; 95% CI: 95% confidence interval; R^2^: coefficient of determination. Bold values indicate a statistically significant result (*p* < 0.05). NRP1: Neuropilin-1; PLXNA1: Plexin-A1; SEMA3A: Semaphorin 3A; NRP1/PLXNA1 and SEMA3A/NRP1: exploratory Sema3A-related biomarker ratios; PTH: parathyroid hormone; BMI: body mass index; DM: diabetes mellitus; CV: coefficient of variation. CV coefficients represent the estimated change per 1-percentage-point increase in CV. Variance inflation factors and tolerance values for all covariates (identical across models) are reported in Supplementary Table S6.

## Data Availability

The data supporting the findings of this study are available from the corresponding author upon reasonable request. The data are not publicly available due to privacy and ethical restrictions related to patient information.

## Supplementary Materials

The following supporting information can be downloaded at https://www.mdpi.com/article/10.3390/metabo16100723/s1, Table S1: Other laboratory values of the patients (according to glycemic control groups); Table S2: Medication use profile of the patients by glycemic control group (*n* = 126) [revised]; Table S3: Quartile-Based analysis of bone turnover indices across Δ glucose quartiles (*n* = 126); Table S4: Full Spearman correlation results with Benjamini–Hochberg FDR-adjusted q-values; Table S5: ELISA assay specifications for bone turnover and Sema3A-related biomarkers; Table S6: Multicollinearity diagnostics, standardized coefficients, and sensitivity analyses for glycemic variability indices; Table S7: Baseline characteristics of included (*n* = 126) versus excluded (*n* = 13) patients; Table S8: Absolute bone mineral density (g/cm^2^) by glycemic control group; Figure S1: Distribution of bone turnover indices across glycemic variability quartiles.

## Abbreviations

The following abbreviations are used in this manuscript:

T2DM	Type 2 diabetes mellitus
BMD	Bone mineral density
DXA	Dual-energy X-ray absorptiometry
HbA1c	Hemoglobin A1c
FPG	Fasting plasma glucose
BMI	Body mass index
GV	Glycemic variability
SD	Standard deviation
CV	Coefficient of variation
P1NP	Procollagen type 1 N-terminal propeptide
CTX-1	C-terminal telopeptide of type 1 collagen
Sema3A	Semaphorin 3A
NRP1	Neuropilin-1
PLXNA1	Plexin-A1
PTH	Parathyroid hormone
TBS	Trabecular bone score
ELISA	Enzyme-linked immunosorbent assay
WHO	World Health Organization
IDF	International Diabetes Federation
FDR	False discovery rate
CGM	Continuous glucose monitoring
VIF	Variance inflation factor
