# Supplementary material for: Association Between Glycemic Variability and Bone Turnover and the Sema3A Signaling Pathway in Postmenopausal Women with Type 2 Diabetes Mellitus
Source: Metabolites. 2026 Sep 28;16(10):723. doi: 10.3390/metabo16100723 (PMC13706936; doi:10.3390/metabo16100723)
Supplement: Supplementary file 1 [file metabolites-16-00723-s001.zip › metabolites-4545696-supplementary.pdf]

Supplementary Materials

Association Between Glycemic Variability and Bone Turnover and the Sema3A Signaling Pathway in Postmenopausal Women with Type 2 Diabetes Mellitus

Supplementary Table S1. Other Laboratory Values of the Patients (According to Glycemic Control Groups)

Variable	All patients (n=126)	Group 1 (n=33)	Group 2 (n=47)	Group 3 (n=46)	p value
WBC (x10³/µL)	7.63 [6.74-8.82]	7.62 [6.39-8.68]	8.24 [6.88-8.66]	7.20 [6.79-9.10]	0.510
Platelets (x10³/µL)	259.00 [220.25-311.75]	255.00 [219.00-307.00]	260.00 [225.00-308.50]	264.00 [222.75-328.00]	0.667
Hemoglobin (g/dL)	13.80 [12.90-14.70]	13.80 [12.90-14.70]	13.80 [12.95-14.75]	13.95 [12.95-14.67]	0.995
Urea (mg/dL)	33.00 [27.25-39.00]	32.00 [29.00-37.00]	35.00 [28.00-43.00]	31.00 [27.00-38.00]	0.291
BUN (mg/dL)	15.00 [12.83-18.08]	15.00 [13.80-17.10]	16.00 [13.00-19.75]	14.35 [12.43-17.98]	0.299
Creatinine (mg/dL)	0.72 [0.66-0.83]	0.73 [0.67-0.80]	0.72 [0.66-0.85]	0.70 [0.66-0.83]	0.910
Uric acid (mg/dL)	4.60 [3.70-5.30]	4.90 [4.20-5.70]	4.30 [3.60-5.10]	4.45 [3.52-5.05]	0.046
AST (U/L)	18.00 [16.00-23.00]	19.00 [17.00-23.00]	19.00 [16.50-23.00]	17.70 [15.00-20.75]	0.230
ALT (U/L)	19.00 [15.00-24.00]	18.00 [14.00-23.00]	20.00 [15.50-25.00]	17.50 [15.00-22.00]	0.412
GGT (U/L)	20.00 [15.25-26.00]	20.00 [15.00-24.00]	20.00 [16.00-26.00]	20.00 [15.00-29.75]	0.489
ALP (U/L)	87.00 [72.00-105.00]	80.00 [69.00-106.00]	85.00 [72.00-103.50]	91.00 [76.00-105.00]	0.364
Albumin (g/dL)	4.50 [4.30-4.60]	4.50 [4.20-4.60]	4.50 [4.35-4.60]	4.40 [4.20-4.60]	0.190
Calcium (mg/dL)	9.70 [9.43-9.90]	9.60 [9.30-9.80]	9.70 [9.50-9.95]	9.70 [9.50-10.00]	0.344
Phosphorus (mg/dL)	3.70 [3.30-4.10]	3.50 [3.30-3.80]	3.80 [3.20-4.10]	3.70 [3.40-4.10]	0.313
Magnesium (mg/dL)	1.90 [1.70-2.10]	1.90 [1.80-2.10]	1.90 [1.70-2.00]	1.90 [1.80-2.00]	0.477
TSH (µIU/mL)	2.20 [1.49-2.98]	2.17 [1.36-2.84]	2.10 [1.49-3.11]	2.27 [1.57-2.98]	0.714

Data are presented as median [interquartile range]. The Kruskal-Wallis test was used for the three-group comparison. Group 1: HbA1c 5-7%; Group 2: HbA1c 7-9%; Group 3: HbA1c ≥9%. WBC: white blood cell count; BUN: blood urea nitrogen; AST: aspartate aminotransferase; ALT: alanine aminotransferase; GGT: gamma-glutamyl transferase; ALP: alkaline phosphatase; TSH: thyroid-stimulating hormone.

Supplementary Table S2. Medication Use Profile of the Patients by Glycemic Control Group (n=126) [REVISED]

Medication	Group 1 (n=33)	Group 2 (n=47)	Group 3 (n=46)	p value
Insulin use	8/33 (24.2%)	27/47 (57.4%)	33/46 (71.7%)	<0.001
Basal insulin	8/33 (24.2%)	27/47 (57.4%)	33/46 (71.7%)	<0.001
Bolus insulin	5/33 (15.2%)	22/47 (46.8%)	25/46 (54.3%)	0.001
Metformin	31/33 (93.9%)	33/47 (70.2%)	35/46 (76.1%)	0.034
SGLT2 inhibitor (total)	19/33 (57.6%)	40/47 (85.1%)	41/46 (89.1%)	0.001
Dapagliflozin	13/32 (40.6%)	30/47 (63.8%)	30/46 (65.2%)	0.060
Empagliflozin	6/33 (18.2%)	10/47 (21.3%)	11/46 (23.9%)	0.829
DPP-4 inhibitor	16/33 (48.5%)	33/47 (70.2%)	33/46 (71.7%)	0.066
Sulfonylurea	5/33 (15.2%)	11/47 (23.4%)	11/46 (23.9%)	0.592
Nateglinide	0/33 (0.0%)	3/47 (6.4%)	1/46 (2.2%)	0.246
Acarbose	0/33 (0.0%)	0/46 (0.0%)	2/46 (4.3%)	0.175
Pioglitazone	2/33 (6.1%)	3/46 (6.5%)	3/46 (6.5%)	0.996
Statin	15/33 (45.5%)	31/47 (66.0%)	31/46 (67.4%)	0.099
Fenofibrate	0/33 (0.0%)	3/47 (6.4%)	3/46 (6.5%)	0.327
Ezetimibe	0/33 (0.0%)	0/47 (0.0%)	1/46 (2.2%)	0.416
RAS blocker (total)	23/33 (69.7%)	32/47 (68.1%)	27/46 (58.7%)	0.517
ACE inhibitor	7/33 (21.2%)	11/47 (23.4%)	9/46 (19.6%)	0.903
ARB	16/33 (48.5%)	21/47 (44.7%)	18/46 (39.1%)	0.699
Thiazide	11/33 (33.3%)	13/47 (27.7%)	14/46 (30.4%)	0.861
Indapamide	3/32 (9.4%)	4/47 (8.5%)	4/46 (8.7%)	0.991
Calcium channel blocker	16/33 (48.5%)	23/47 (48.9%)	14/46 (30.4%)	0.134
Beta blocker	14/33 (42.4%)	12/47 (25.5%)	10/46 (21.7%)	0.113
Aldosterone antagonist	0/33 (0.0%)	0/47 (0.0%)	2/46 (4.3%)	0.171
Alpha blocker	0/33 (0.0%)	0/47 (0.0%)	1/46 (2.2%)	0.416
ASA	11/33 (33.3%)	16/47 (34.0%)	14/46 (30.4%)	0.927

Data are presented as n/N (%). Categories are not mutually exclusive; patients may be included in more than one medication group. Groups compared with the chi-square test (Fisher's exact test noted where expected cell counts were low). Group 1: HbA1c 5-7%; Group 2: HbA1c 7-9%; Group 3: HbA1c ≥9%. RAS: renin-angiotensin system; SGLT2: sodium-glucose cotransporter-2. This table replaces the previous version, which reported only overall (non-group-stratified) medication frequencies.

Supplementary Table S3. Quartile-Based Analysis of Bone Turnover Indices Across Δ Glucose Quartiles (n=126)

Variable	Q1 (<96.2 mg/dL, n=32)	Q2 (96.2-134.8 mg/dL, n=31)	Q3 (134.8-184.5 mg/dL, n=31)	Q4 (≥184.5 mg/dL, n=32)	p (K-W)	r (trend)
P1NP/CTX-1	0.097 [0.082-0.147]	0.103 [0.080-0.156]	0.076 [0.069-0.103]	0.073 [0.062-0.088]	<0.001	-0.368
NRP1/PLXNA1	0.124 [0.095-0.162]	0.114 [0.096-0.139]	0.095 [0.088-0.118]	0.082 [0.074-0.095]	<0.001	-0.501
SEMA3A/NRP1	0.116 [0.053-0.131]	0.118 [0.052-0.140]	0.127 [0.076-0.140]	0.136 [0.112-0.151]	0.096	+0.249

Data are presented as median [interquartile range]. The Kruskal-Wallis test (p K-W) was used for group comparison; these quartile groupings are sample-derived and exploratory, not validated clinical thresholds. r (trend): Spearman trend correlation coefficient calculated using Δ Glucose as a continuous variable. P1NP/CTX-1: bone turnover formation/resorption coupling ratio; NRP1/PLXNA1 and SEMA3A/NRP1: exploratory Sema3A-related biomarker ratios; K-W: Kruskal-Wallis test.

Supplementary Table S4. Full Spearman Correlation Results with Benjamini–Hochberg FDR-Adjusted q-Values (55 Correlations, n=126)

Outcome	Glycemic variability parameter	Spearman r	Raw p	FDR q	Significant at q<0.05
P1NP	Mean glucose	-0.141	0.114	0.233	No
P1NP	SD	-0.281	0.001	0.006	Yes
P1NP	CV	-0.272	0.002	0.008	Yes
P1NP	Δ Glucose	-0.288	0.001	0.005	Yes
P1NP	Postprandial increment	-0.173	0.052	0.131	No
CTX-1	Mean glucose	+0.143	0.109	0.231	No
CTX-1	SD	+0.287	0.001	0.005	Yes
CTX-1	CV	+0.314	<0.001	0.002	Yes
CTX-1	Δ Glucose	+0.205	0.022	0.059	No
CTX-1	Postprandial increment	+0.126	0.158	0.290	No
NRP1	Mean glucose	-0.052	0.562	0.672	No
NRP1	SD	-0.210	0.018	0.056	No
NRP1	CV	-0.214	0.016	0.052	No
NRP1	Δ Glucose	-0.331	<0.001	<0.001	Yes
NRP1	Postprandial increment	-0.072	0.426	0.558	No
PLXNA1	Mean glucose	+0.104	0.247	0.367	No
PLXNA1	SD	+0.333	<0.001	<0.001	Yes
PLXNA1	CV	+0.403	<0.001	<0.001	Yes
PLXNA1	Δ Glucose	+0.228	0.010	0.035	Yes
PLXNA1	Postprandial increment	+0.167	0.062	0.142	No
SEMA3A	Mean glucose	-0.086	0.340	0.467	No
SEMA3A	SD	+0.020	0.827	0.891	No
SEMA3A	CV	+0.028	0.759	0.852	No
SEMA3A	Δ Glucose	+0.016	0.858	0.891	No
SEMA3A	Postprandial increment	-0.081	0.365	0.490	No
P1NP/CTX-1	Mean glucose	-0.135	0.132	0.260	No
P1NP/CTX-1	SD	-0.395	<0.001	<0.001	Yes
P1NP/CTX-1	CV	-0.415	<0.001	<0.001	Yes
P1NP/CTX-1	Δ Glucose	-0.368	<0.001	<0.001	Yes
P1NP/CTX-1	Postprandial increment	-0.171	0.055	0.132	No
NRP1/PLXNA1	Mean glucose	-0.117	0.193	0.327	No
NRP1/PLXNA1	SD	-0.451	<0.001	<0.001	Yes
NRP1/PLXNA1	CV	-0.490	<0.001	<0.001	Yes
NRP1/PLXNA1	Δ Glucose	-0.501	<0.001	<0.001	Yes
NRP1/PLXNA1	Postprandial increment	-0.144	0.107	0.231	No
SEMA3A/NRP1	Mean glucose	-0.025	0.784	0.862	No
SEMA3A/NRP1	SD	+0.190	0.033	0.086	No
SEMA3A/NRP1	CV	+0.205	0.021	0.059	No
SEMA3A/NRP1	Δ Glucose	+0.249	0.005	0.018	Yes
SEMA3A/NRP1	Postprandial increment	-0.008	0.932	0.949	No
L1-L4 T-score	Mean glucose	-0.056	0.532	0.651	No
L1-L4 T-score	SD	-0.105	0.241	0.367	No
L1-L4 T-score	CV	-0.058	0.517	0.646	No
L1-L4 T-score	Δ Glucose	-0.090	0.315	0.445	No
L1-L4 T-score	Postprandial increment	-0.098	0.274	0.397	No
Femoral neck T-score	Mean glucose	-0.114	0.202	0.327	No
Femoral neck T-score	SD	-0.121	0.177	0.314	No
Femoral neck T-score	CV	-0.130	0.146	0.276	No

Outcome	Glycemic variability parameter	Spearman r	Raw p	FDR q	Significant at q<0.05
Femoral neck T-score	Δ Glucose	-0.069	0.442	0.565	No
Femoral neck T-score	Postprandial increment	-0.115	0.198	0.327	No
Total femur T-score	Mean glucose	-0.017	0.850	0.891	No
Total femur T-score	SD	+0.042	0.640	0.749	No
Total femur T-score	CV	+0.002	0.980	0.980	No
Total femur T-score	Δ Glucose	+0.037	0.683	0.783	No
Total femur T-score	Postprandial increment	-0.105	0.242	0.367	No

Benjamini–Hochberg false discovery rate (FDR) correction applied across all 55 correlations. Of 21 correlations significant at raw p<0.05, 16 remained significant at FDR q<0.05; five (NRP1–CV, NRP1–SD, SEMA3A/NRP1–CV, CTX-1–Δ glucose, SEMA3A/NRP1–SD) did not survive correction. This table was independently recomputed from the raw study dataset and reproduces the summary reported in the main-text Table 3 footnote exactly.

Supplementary Table S5. ELISA Assay Specifications for Bone Turnover and Sema3A-Related Biomarkers

Biomarker	Catalog no.	Sensitivity	Standard curve range	Intra-/inter-assay CV
P1NP	E1350Hu	1.15 ng/mL	2.5–160 ng/mL	<8% / <10%
CTX-1	E1349Hu	4.21 ng/mL	7–1500 ng/mL	<8% / <10%
SEMA3A	E2078Hu	0.024 ng/mL	0.05–20 ng/mL	<8% / <10%
PLXNA1	E2631Hu	10.14 ng/mL	20–3500 ng/mL	<8% / <10%
NRP1	E2101Hu	0.054 ng/mL	0.1–40 ng/mL	<8% / <10%

All kits: BT Lab (Bioassay Technology Laboratory, Jiaxing, Zhejiang, China), double-antibody sandwich ELISA. Assay procedure followed the manufacturer's protocol (biotinylated antibody, then Streptavidin-HRP conjugate, TMB substrate, 37°C dark incubation); optical density read at 450 nm on a Thermo Scientific Multiskan SkyHigh microplate reader (Thermo Fisher Scientific, Vantaa, Finland). All samples were run in a single batch, in duplicate; intra- and inter-assay coefficients of variation were calculated for each measurement. Independent validation of these kits against international reference methods is limited, as noted in the manuscript's Limitations section. P1NP: procollagen type 1 N-terminal propeptide; CTX-1: C-terminal telopeptide type 1; SEMA3A: Semaphorin 3A; PLXNA1: Plexin A1; NRP1: Neuropilin 1; CV: coefficient of variation.

Supplementary Table S6. Multicollinearity Diagnostics, Standardized Coefficients, and Sensitivity Analyses for Glycemic Variability Indices

S6a. Variance Inflation Factors (VIF) and Tolerance

Covariate	VIF	Tolerance
Age	1.20	0.830
BMI	1.05	0.952
DM duration	1.34	0.745
Vitamin D	1.15	0.871
PTH	1.06	0.940
Insulin use	1.41	0.708
Mean glucose	1.51	0.661
CV (%)	2.43	0.412
Δ Glucose	2.96	0.338

Covariate set is identical across all seven regression models (Tables 3 and 4), so VIF/tolerance values apply to all models. All VIF values <5, confirming the absence of problematic multicollinearity; CV and Δ glucose show the highest (but still acceptable) VIF, consistent with their shared derivation from the same capillary glucose measurements.

S6b. Standardized (β) Coefficients for CV and Δ Glucose in the Mutually Adjusted Models

Outcome	CV standardized β	CV p	Δ Glucose standardized β	Δ Glucose p
P1NP/CTX-1	-0.298	0.017	-0.119	0.382
P1NP	-0.154	0.234	-0.159	0.266
CTX-1	+0.211	0.140	-0.111	0.480
NRP1/PLXNA1	-0.136	0.286	-0.404	0.005
SEMA3A/NRP1	-0.086	0.504	+0.552	<0.001
PLXNA1	+0.159	0.262	+0.098	0.528
NRP1	-0.010	0.941	-0.372	0.013

Standardized coefficients obtained by z-standardizing all model variables before regression; p-values are identical to the unstandardized models in Tables 3 and 4.

S6c. Single-Metric Models versus the Mutually Adjusted Model (CV and Δ Glucose)

Outcome	GV metric	B, single-metric model*	p	B, mutually adjusted model†	p
P1NP/CTX-1	CV	-0.00219	<0.001	-0.00174	0.017
P1NP/CTX-1	Δ Glucose	-0.00025	0.001	-0.00008	0.382
P1NP	CV	-0.807	0.006	-0.487	0.234
P1NP	Δ Glucose	-0.108	0.007	-0.061	0.266
CTX-1	CV	+2.654	0.165	+3.999	0.140
CTX-1	Δ Glucose	+0.124	0.634	-0.258	0.480
NRP1/PLXNA1	CV	-0.00187	<0.001	-0.00064	0.286
NRP1/PLXNA1	Δ Glucose	-0.00030	<0.001	-0.00023	0.005
SEMA3A/NRP1	CV	+0.00139	0.006	-0.00045	0.504
SEMA3A/NRP1	Δ Glucose	+0.00031	<0.001	+0.00035	<0.001
PLXNA1	CV	+7.390	0.028	+5.294	0.262
PLXNA1	Δ Glucose	+0.906	0.046	+0.401	0.528
NRP1	CV	-1.115	0.012	-0.045	0.941
NRP1	Δ Glucose	-0.209	<0.001	-0.205	0.013

**Single-metric models include mean glucose plus the named GV metric only (age, BMI, DM duration, vitamin D, PTH, and insulin use included in all models); the competing GV metric (CV or Δ glucose) is excluded. †Mutually adjusted model is the model reported in Tables 3 and 4 (mean glucose, CV, and Δ glucose entered simultaneously). Each GV metric is broadly significant when modeled alone, but only one metric per outcome retains significance once both are entered together — the expected signature of shared variance between two indices derived from the same capillary glucose measurements, not a data inconsistency.*

S6d. Sensitivity Analyses: Excluding Pioglitazone Users and Adjusting for SGLT2 Inhibitor Use

Outcome	GV metric	B, pioglitazone excluded (n=116)	p	B, SGLT2i-adjusted (n=125)	p
P1NP/CTX-1	CV	-0.00196	0.011	-0.00175	0.017
P1NP/CTX-1	Δ Glucose	-0.00006	0.568	-0.00008	0.390
P1NP	CV	-0.558	0.194	-0.500	0.217
P1NP	Δ Glucose	-0.051	0.374	-0.058	0.285
CTX-1	CV	+4.357	0.115	+3.919	0.145
CTX-1	Δ Glucose	-0.284	0.441	-0.240	0.507
NRP1/PLXNA1	CV	-0.00088	0.158	-0.00065	0.281
NRP1/PLXNA1	Δ Glucose	-0.00020	0.017	-0.00023	0.005
SEMA3A/NRP1	CV	-0.00024	0.729	-0.00044	0.515
SEMA3A/NRP1	Δ Glucose	+0.00032	<0.001	+0.00035	<0.001
PLXNA1	CV	+6.406	0.196	+5.219	0.269
PLXNA1	Δ Glucose	+0.301	0.650	+0.418	0.512
NRP1	CV	-0.076	0.904	-0.066	0.912
NRP1	Δ Glucose	-0.194	0.022	-0.200	0.014

Pioglitazone-excluded model: same mutually adjusted model as Tables 3/4, restricted to the 116 participants not receiving pioglitazone. SGLT2i-adjusted model: same mutually adjusted model with SGLT2 inhibitor use (yes/no) added as an additional covariate (n=125); SGLT2 inhibitor use itself was not significantly associated with any outcome (all p>0.05, not tabulated). Primary associations (CV→P1NP/CTX-1; Δ glucose→NRP1/PLXNA1, SEMA3A/NRP1, NRP1) remain materially unchanged in both sensitivity analyses.

S6e. Log-Transformed Ratio Sensitivity Models

Outcome (ratio)	Model	B (Δ glucose or CV, as in Tables 3–4)	p
P1NP/CTX-1 (n=125)	Untransformed	-0.00174	0.017
	Log-transformed [ln(P1NP)−ln(CTX-1)]	-0.02062	0.001
NRP1/PLXNA1 (n=125)	Untransformed	-0.00023	0.005
	Log-transformed [ln(NRP1)−ln(PLXNA1)]	-0.00233	<0.001
SEMA3A/NRP1 (n=125)	Untransformed	+0.00035	<0.001
	Log-transformed [ln(SEMA3A)−ln(NRP1)]	+0.00365	0.001

Distributions of the raw ratios were right-skewed for P1NP/CTX-1 (skewness=1.84) and NRP1/PLXNA1 (skewness=1.68), and approximately symmetric for SEMA3A/NRP1 (skewness=-0.21). To assess whether the associations reported in Tables 3–4 reflect a denominator-driven artifact of the untransformed ratios, each ratio was re-modeled in log form [ln(numerator)−ln(denominator)], using the same covariates as the corresponding mutually adjusted model. The glycemic-variability coefficient (CV for P1NP/CTX-1; Δ glucose for NRP1/PLXNA1 and SEMA3A/NRP1) remained significant, and in each case slightly stronger, in the log-transformed model, indicating that these associations are not artifacts of ratio scaling. Component-level models (P1NP, CTX-1, NRP1, PLXNA1 individually) are already reported alongside the ratio models in Tables 3–4.

S6f. Sensitivity Analysis Adjusting for HbA1c as a Continuous Variable

Outcome	GV metric	B, primary model (Tables 3–4)	p	B, HbA1c-adjusted model	p
P1NP/CTX-1	CV	-0.1743	0.017	-0.1767	0.016
	Δ Glucose	-0.00008	0.382	-0.00008	0.416
P1NP	CV	-48.686	0.234	-50.127	0.222
	Δ Glucose	-0.0614	0.266	-0.0582	0.296
CTX-1	CV	399.89	0.140	393.28	0.148
	Δ Glucose	-0.2575	0.480	-0.2428	0.509
NRP1/PLXNA1	CV	-0.0645	0.286	-0.0677	0.264
	Δ Glucose	-0.00023	0.005	-0.00023	0.006
SEMA3A/NRP1	CV	-0.0446	0.504	-0.0408	0.542
	Δ Glucose	0.00035	<0.001	0.00034	<0.001
PLXNA1	CV	529.45	0.262	527.86	0.266
	Δ Glucose	0.4012	0.528	0.4047	0.528
NRP1	CV	-4.482	0.941	-7.712	0.899
	Δ Glucose	-0.2049	0.013	-0.1977	0.017

HbA1c-adjusted model: same mutually adjusted model as Tables 3–4 (age, BMI, DM duration, vitamin D, PTH, insulin use, mean glucose, CV, and Δ glucose), with HbA1c (%) added as an additional continuous covariate. In this model, HbA1c itself was not significantly associated with any outcome (P1NP/CTX-1: B=-0.00225, p=0.571; P1NP: B=-1.344, p=0.550; CTX-1: B=-6.167, p=0.678; NRP1/PLXNA1: B=-0.00305, p=0.357; SEMA3A/NRP1: B=0.00355, p=0.333; PLXNA1: B=-1.480, p=0.955; NRP1: B=-3.011, p=0.364), and the coefficients for CV and Δ glucose were materially unchanged from the primary models, indicating that the reported glycemic-variability associations are not attributable to, or confounded by, longer-term average glycemic exposure as captured by HbA1c.

Supplementary Table S7. Baseline Characteristics of Included (n=126) versus Excluded (n=13) Patients

Variable	Included (n=126)	Excluded (n=13)	p
Age (years)	64.0 [59.0–67.8]	63.0 [58.0–68.0]	0.698
T2DM duration (years)	13.0 [10.0–15.0]	14.0 [10.0–17.0]	0.585
BMI (kg/m²)	28.7 [28.0–29.2]	28.9 [28.5–29.1]	0.811
Fasting plasma glucose (mg/dL)	158.5 [126.0–204.8]	182.0 [120.0–227.0]	0.748
Total cholesterol (mg/dL)	190.0 [160.5–213.2]	181.0 [156.0–221.0]	0.928
LDL-C (mg/dL)	103.0 [77.0–128.0]	87.0 [75.0–126.0]	0.580
HDL-C (mg/dL)	49.0 [41.0–57.0]	48.0 [41.0–66.0]	0.492
Triglyceride (mg/dL)	159.5 [123.5–216.8]	200.0 [119.0–248.0]	0.424
Insulin use, n (%)	68 (54.0%)	5 (38.5%)	0.384
Hypertension, n (%)	94 (74.6%)	4 (30.8%)	0.002
Hyperlipidemia, n (%)	79 (62.7%)	5 (38.5%)	0.134
Coronary artery disease, n (%)	22 (17.5%)	6 (46.2%)	0.024

Continuous variables are presented as median [interquartile range] and compared using the Mann-Whitney U test; categorical variables are presented as n (%) and compared using Fisher's exact test. Serum creatinine and hemoglobin could not be reliably compared because of apparent data-entry/formatting inconsistencies affecting these two fields for several excluded patients in the source dataset; all other baseline variables shown were complete for both groups. Baseline demographic and metabolic profiles were comparable between included and excluded patients, but excluded patients had a lower prevalence of hypertension and a higher prevalence of coronary artery disease, indicating that exclusion was not entirely independent of comorbidity burden. BMI: body mass index; LDL-C: low-density lipoprotein cholesterol; HDL-C: high-density lipoprotein cholesterol; T2DM: type 2 diabetes mellitus.

Supplementary Table S8. Absolute Bone Mineral Density (g/cm²) by Glycemic Control Group

Site	Overall (n=126)	Group 1 (n=33)	Group 2 (n=47)	Group 3 (n=46)	p
L1–L4 BMD (g/cm²)	0.942 [0.814–1.036]	0.981 [0.853–1.039]	0.901 [0.797–1.063]	0.929 [0.815–1.013]	0.404
Femoral neck BMD (g/cm²)	0.796 [0.714–0.876]	0.799 [0.713–0.872]	0.792 [0.731–0.871]	0.811 [0.709–0.897]	0.924
Total femur BMD (g/cm²)	0.893 [0.830–0.990]	0.884 [0.840–0.984]	0.900 [0.821–0.987]	0.905 [0.832–1.004]	0.915

Values are median [interquartile range]; groups compared using the Kruskal-Wallis test. Absolute BMD did not differ significantly across HbA1c-based glycemic control groups at any site, consistent with the T-score results reported in Table 1. BMD: bone mineral density.

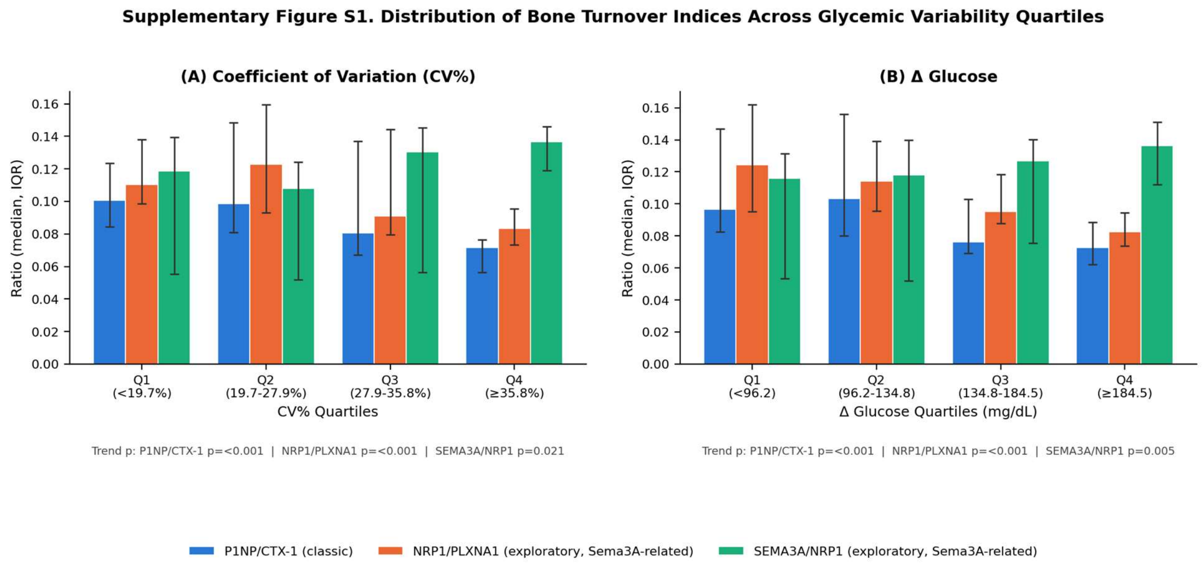

Supplementary Figure S1. Distribution of Bone Turnover Indices Across Glycemic Variability Quartiles.

Median (interquartile range) values of the P1NP/CTX-1 (classic bone turnover coupling index), NRP1/PLXNA1, and SEMA3A/NRP1 (exploratory Sema3A-related biomarker ratios) across quartiles of (A) coefficient of variation (CV%) and (B) Δ glucose. Trend p-values were derived from Spearman correlation using the respective glycemic variability parameter as a continuous variable; these quartile groupings are sample-derived and exploratory, not validated clinical thresholds. Error bars represent the interquartile range. P1NP: procollagen type 1 N-terminal propeptide; CTX-1: C-terminal telopeptide type 1; NRP1: Neuropilin-1; PLXNA1: Plexin-A1; SEMA3A: Semaphorin 3A.
